# Expression of the Lhx genes *apterous* and *lim1* in an errant polychaete: implications for bilaterian appendage evolution, neural development, and muscle diversification

**DOI:** 10.1186/2041-9139-4-4

**Published:** 2013-02-01

**Authors:** Christopher J Winchell, David K Jacobs

**Affiliations:** 1 Department of Ecology and Evolutionary Biology, University of California, Los Angeles, 621 Charles E Young Drive South, Los Angeles, CA 90095-1606, USA; 2 Present address: Department of Molecular and Cell Biology, University of California, Berkeley, 515 LSA #3200, Berkeley, CA 94720-3200, USA

## Abstract

**Background:**

Arthropod and vertebrate appendages appear to have evolved via parallel co-option of a plesiomorphic gene regulatory network. Our previous work implies that annelids evolved unrelated appendage-forming mechanisms; we therefore found no support for homology of parapodia and arthropodia at the level of the whole appendage. We expand on that study here by asking whether expression of the LIM homeobox (Lhx) genes *apterous* and *lim1* in the annelid *Neanthes arenaceodentata* supports homology of the dorsal branches as well as the proximodistal axes of parapodia and arthropodia. In addition, we explore whether the neural expression of *apterous* and *lim1* in *Neanthes* supports the putative ancestral function of the Lhx gene family in regulating the differentiation and maintenance of neuronal subtypes.

**Results:**

Both genes exhibit continuous expression in specific portions of the developing central nervous system, from hatching to at least the 13-chaetiger stage. For example, nerve cord expression occurs in segmentally iterated patterns consisting of diffuse sets of many *lim1*-positive cells and comparatively fewer, clustered pairs of *apterous*-positive cells. Additionally, continuous *apterous* expression is observed in presumed neurosecretory ganglia of the posterior brain, while *lim1* is continuously expressed in stomatogastric ganglia of the anterior brain. *apterous* is also expressed in the jaw sacs, dorsal parapodial muscles, and a presumed pair of cephalic sensory organs, whereas *lim1* is expressed in multiple pharyngeal ganglia, the segmental peripheral nervous system, neuropodial chaetal sac muscles, and parapodial ligules.

**Conclusions:**

The early and persistent nervous system expression of *apterous* and *lim1* in *Neanthes* juveniles supports conservation of Lhx function in bilaterian neural differentiation and maintenance. Our results also suggest that diversification of parapodial muscle precursors involves a complementary LIM code similar to those generating distinct neuronal identities in fly and mouse nerve cords. Expression of *apterous* and *lim1* in discrete components of developing parapodia is intriguing but does not map to comparable expression of these genes in developing arthropod appendages. Thus, annelid and arthropod appendage development apparently evolved, in part, via distinct co-option of the neuronal regulatory architecture. These divergent patterns of *apterous* and *lim1* activity seemingly reflect *de novo* origins of parapodia and arthropodia, although we discuss alternative hypotheses.

## Background

Annelid parapodia and arthropod legs (arthropodia) are well-known types of protostome appendages. Under the Articulata hypothesis, which posits an annelid-arthropod sister relationship, parapodia and arthropodia are often considered homologous [1]. However, favor for this notion has waned considerably since the late 1990s [2,3], as new types of phylogenetic data and new views of animal relationships have become established (see [4] for a recent review). Despite the firm molecular-phylogenetic separation of annelids from arthropods, and the paucity of morphologic details to homologize their appendages, the evolutionary relationship between parapodia and arthropodia is still a contentious issue, with some authors strongly favoring (for example, [5]), guardedly favoring, (for example, [6]), or contradicting (for example, [7]) homology.

Previously, we addressed this controversy from a developmental-genetic perspective by analyzing the expression of *Distalless*, *dachshund*, and *optomotor blind* (three arthropod/vertebrate appendage genes) in the parapodia-bearing polychaete *Neanthes arenaceodentata*[8]. Finding no compelling evidence of shared developmental mechanisms of appendage formation, we concluded that parapodia evolved independently of arthropodia and vertebrate limbs. These previous results are intriguing because similar mechanisms *do* control certain aspects of arthropod and vertebrate appendage development [9-11], suggesting that a primitive regulatory network (present in the protostome-deuterostome ancestor) underwent parallel recruitment to appendage-forming roles in arthropods and vertebrates, but not in annelids. We revisit this issue in the current paper by examining whether the expression of two additional *Neanthes* genes, orthologs of the LIM homeobox (Lhx) genes *apterous* (*ap*) and *lim1*, provide evidence for or against homology of particular architectural features of annelid and arthropod appendages.

In diverse arthropods, expression patterns of *ap* and the POU homeobox gene *pdm* suggest that a common ancestor bore branched limbs with distinct dorsal and ventral elements. In crustaceans, for example, *ap* and *pdm* are expressed throughout the developing gill-like epipods that branch dorsally from the limbs [12,13]. Evidence from the fruit fly *Drosophila melanogaster* suggests that insect wings are also akin to dorsal elements of branched limbs, as they originate from common embryonic primordia shared with legs [14,15], and indeed, *ap* and *pdm* are expressed in developing *Drosophila* wings, where *ap* specifies the dorsal compartment, and *pdm* specifies a proximodistal growth center [16-19] (note that *ap* is also required for proper leg development: its loss of function leads to deletion of the fourth tarsal segment [20]). Furthermore, because *ap* and *pdm* are expressed in the developing book gills, book lungs, tubular tracheae, and spinnerets of chelicerates, it has been argued that these opisthosomal structures, like crustacean gills and insect wings, were modified from the dorsal elements of ancestral arthropod limbs, and that shared ancestral ventral elements (legs) were altogether lost from the chelicerate opisthosoma [13]. This apparently branched, plesiomorphic limb architecture for arthropods, coupled with the fact that the parapodia of certain polychaetes, like those of *Neanthes*, are conspicuously branched appendages bearing dorsal (notopodial) and ventral (neuropodial) divisions, raises the question as to whether this trait is ancestral for protostomes. If annelids and arthropods inherited a homologous limb architecture with a pronounced dorsal-ventral bifurcation, then we would expect *ap* expression in *Neanthes* to occur extensively throughout developing notopodia.

The expression and function of *lim1* in arthropod appendages has so far been examined in only *Drosophila* and the red flour beetle *Tribolium castaneum*, but it is required in both of these insects for proper proximodistal patterning. In *Drosophila*, *lim1* expression in leg imaginal discs corresponds to the coxal, femoral, lower tibial, and pretarsal segments. Its transcripts are also present in the first and second segments and aristae of developing antennae. These portions of the legs and antennae are lacking or severely deformed in *lim1* loss-of-function mutants [20,21]. The *lim1* ortholog of *Tribolium* is expressed in proximal, middle, and distal domains within developing legs [22], as well as in antennal and gnathal appendages prior to their outgrowth [23]. Knockdown of *Tribolium lim1* by RNA interference produces malformed appendages, usually with shortened and fused segments [23-25]. Because many aspects of appendage patterning appear to be conserved in general across diverse arthropods [26-29], and because mechanisms of proximodistal leg patterning may even be conserved throughout the Panarthropoda [30], it is reasonable to anticipate a role for *lim1* in arthropod proximodistal pattering outside of insects, and to select *lim1* as a gene of interest in the study of protostome appendage evo-devo. If the proximodistal axes of arthropodia and parapodia are homologous, then we expect *lim1* expression in *Neanthes* to reflect a role in the patterning of this axis.

In addition to inferring the evolutionary relationship between parapodia and arthropodia, we analyze *ap* and *lim1* expression in *Neanthes* to explore their involvement in lophotrochozoan neural differentiation. Studies of Lhx genes in model ecdysozoans and deuterostomes suggest this gene family specified and maintained neural subtypes and regulated the targeting of axonal projections in ancestral bilaterians [31]; a comparison of *ap* and *lim1* function in fly and vertebrates illustrates this putative conservation. In the central nervous system of *Drosophila*, *ap* controls axonal pathfinding and fasciculation of particular interneurons [32,33], and it establishes and maintains the identities of FMRF-amide- and leucokinin-producing neurons [34,35]. Similarly, the vertebrate *ap* orthologs *Lhx2* and *Lhx9* confer the rostral orientation of axonal projections of particular spinal cord interneurons [36], and their expression in vertebrate brains is consistent with overlapping and distinct roles in specifying and maintaining particular neural identities [37-39]. Suggesting conservation of olfactory development and physiology, *Lhx2* is required for olfactory sensory neuron identity in vertebrates [40], and *ap* is expressed in adult fly olfactory organs [41].

As in *ap*, *Drosophila lim1* is responsible for guiding neuronal processes to their targets; an example is its control over the dendritic growth of projection neurons to antennal lobe glomeruli [42]. The vertebrate *lim1* orthologs *Lhx1* and *Lhx5* confer the caudal orientation of axonal projections of certain spinal cord interneurons [36]. Although *lim1*’s function in the fly nerve cord is not yet known, it is expressed in a subset of developing interneurons and in motor neurons that innervate dorsal segmental muscles [43]. The latter expression pattern, coupled with the fact that *Lhx1* in vertebrates directs spinal cord motor axons to dorsal limb muscles [44,45], suggests conserved organization of a bilaterian motor circuit. Lastly, mammalian *Lhx1* and *Lhx5* cooperate in assigning identities to post-mitotic neurons, such as Purkinje cells [46] and GABAergic inhibitory neurons [47]. The above observations lead to a prediction of early and persistent *ap* and *lim1* expression in numerous domains of the differentiating central nervous system of *Neanthes*. Furthermore, *ap* and *lim1* domains should be largely distinct from one another, and *ap* expression should occur in developing olfactory organs.

We report detailed observations of *ap* and *lim1* expression during post-embryonic development in *Neanthes*. We find that both genes are expressed in pre-morphogenetic and outgrowing parapodia, but in patterns that are not consistent with the predictions made above. In the nervous system, expression is largely consistent with the putative ancestral role of bilaterian Lhx genes in regulating the differentiation and maintenance of neuronal subtypes. Our observations also suggest a LIM code for parapodial myoblast diversification, as well as instances of co-optive evolution of Lhx function in annelids. For example, *lim1* mRNAs accumulate in parapodial ligules, and *ap* mRNAs are observed in the jaw sacs and in the presumed Langdon’s organs, which are paired cephalic sense organs known only in nereidids.

## Results

### Summary of juvenile development and gross morphology

*N*. *arenaceodentata* is a direct developer whose hatchlings are extremely altricial, possessing only a rudimentary head and a few nascent trunk segments with minute parapodial buds. Although the hatchling nervous system reflects the basic organization of the adult nervous system, it undergoes substantial proliferation and differentiation during the first week of post-embryonic development [48]. Moreover, parapodial morphogenesis and neurogenic processes occur in newly added posterior segments throughout life. Because of these qualities, we felt it was unnecessary to analyze embryonic expression to test whether *ap* and *lim1* potentially regulate parapodial development and neural differentiation/maintenance in this species. We overview the process of juvenile development and the basic anatomy of *N*. *arenaceodentata* in the following paragraph; for more complete descriptions see [48-50].

Embryos and juveniles of this species are brooded by their father within his mucoid tube for approximately 30 days post-fertilization (dpf) (at 21°C). Embryos hatch from their egg capsules at 10 dpf. Hatchlings are teardrop-shaped and typically 450 to 500 μm in length. They possess two to three chaetigers (chaetae-bearing segments), a slightly elongated posterior end, and an anterior mound - the nascent prostomium (the pre-segmental portion of the worm housing the brain). Posterior to the prostomium are four bilateral pairs of small ventrolateral buds. The two posterior-most pairs are nascent parapodia, whereas the two anterior-most pairs are developing anterior cirri, which eventually form long, slender sensory appendages. These cirri derive from segmental tissue, but fuse together to create the achaetous ring that ultimately integrates with the prostomium to form the head. By the mid 3-chaetiger stage (11 dpf), the sensory feeding palps form a pair of ventral buds on the prostomium, and the sensory anal cirri bud forth from the post-segmental pygidium. By the 4-chaetiger stage (13 dpf), the prostomium has enlarged significantly and bears emerging sensory antennae at its anterior terminus. In addition, a recognizable mouth forms on the ventral surface of the worm, immediately posterior to the palps, and several nascent segments with achaetigerous parapodial buds are present between the posterior-most (fourth) chaetiger and the pygidium, which bears a cleft-like anus between the anal cirri. Further juvenile development is characterized by the formation of eyes (by the 5-chaetiger stage; 14 dpf), lengthening of all appendages, emergence of an additional pair of anterior cirri (by the 6-chaetiger stage; 15dpf), and a steady rate of segment addition (approximately 1 chaetiger/day). Yolk stores become depleted near the 20-chaetiger stage (approximately 30 dpf), and so juveniles begin dispersing from the parental tube to feed. Concerning parapodial morphology, the main dorsal division, or notopodium, bears the following processes (from dorsal to ventral): a dorsal cirrus, a dorsal notopodial ligule, a pair of chaetal lobes, and a ventral notopodial ligule. The main ventral division, or neuropodium, bears (from dorsal to ventral) a pair of chaetal lobes, a neuropodial ligule, and a ventral cirrus (see [48] for a discussion on the development sequence of parapodial processes in *Neanthes*). Parapodial cirri are sensory, whereas ligules are chiefly respiratory but apparently also capable of sensation [48,51]. Chaetae emerge from between the chaetal lobes and are anchored basally within an internal chaetal sac ensheathed by muscles that move the sac along an internal support rod (the acicula) to effect chaetal protraction and retraction [52].

### Orthology assessment of the *Neanthes apterous*- and *lim1*-related genes

**Figure 1:**
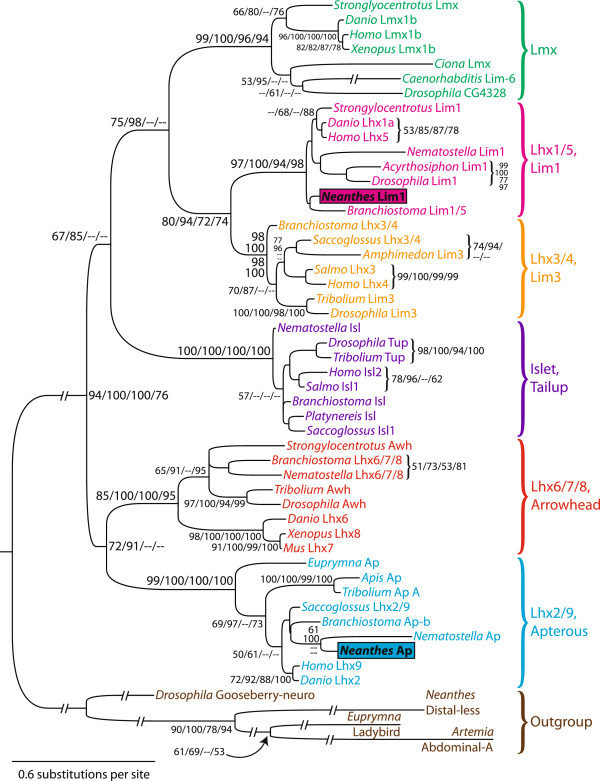
**Maximum Likelihood phylogeny of the LIM-homeodomain family, rooted with four other homeodomain families. **Robust support was found for all subfamilies. The *Neanthes lim1 *and *apterous *orthologs are highlighted with colored boxes. The four support values at each node are, in order: Maximum Likelihood bootstrap percentage, Bayesian posterior probability, Maximum Parsimony bootstrap percentage, and Minimum Evolution bootstrap percentage. Only values ≥50 are shown. Branch lengths are proportional to molecular change (amino acid substitutions/site) between nodes; see scale bar for measurement. Lengths of the interrupted branches were halved to improve the figure’s presentation. Vertebrate subfamily names precede *Drosophila *subfamily names to the right of each bracket. GenBank accession numbers for the analyzed sequences, in order from top to bottom of the tree, are: XP_790548, NP_001020338, NP_002307, NP_001083902, NP_001071756, NP_508204, NP_648567, ACA04473, NP_571291, NP_071758, BAH58087, XP_001945631, NP_572505, AEN75258, ABD59002, XP_002591838, NP_001158395, ACA04748, NP_001130018, EAW91078, XP_973330, NP_724161, XP_001638136, NP_476775, NP_001158279, NP_665804, ACI69553, XP_002609922, ABO93221, NP_001158468, XP_785118, XP_002609417, XP_001626470, XP_971202, NP_523907, NP_001004015, NP_001015899, CAA04012, AAV84105, XP_392622, NP_001139341, NP_001158443, XP_002592485, XP_001635417, AEN75257, NP_001014434, NP_001035099, NP_523862, ACN66454, AAV85467, ABD37012.

The *apterous*-related sequence of *Neanthes* [GenBank: HQ235024] is a 2,027 bp composite of three overlapping gene fragments assembled from 49 clones: 13 for the initial fragment, 14 for the 3′ RACE fragment, and 22 for the 5′ RACE fragment. It contains 318 bp of 5′ UTR (with six in-frame stop codons), a 1,260 bp coding region, and a 449 bp 3′ UTR. The 3,174 bp *lim1*-related sequence [GenBank: HQ235025] was assembled from 44 clones: 12 for the initial fragment, 12 for the 3′ RACE fragment, and 20 for the 5′ RACE fragment. It contains 267 bp of 5′ UTR with two in-frame stop codons, a 1,494 bp coding region, and a 1,413 bp 3′ UTR. To determine affinities of these sequences within the LIM-homeodomain (LIM-HD) family of transcription factors, our phylogenetic analyses included representative sequences of the six canonical LIM-HD subfamilies. We did not include sequences from a possible seventh subfamily, the LIM-only proteins. These molecules lack homeodomains, but recent evidence suggests they originated from within the LIM-HD family after the divergence of sponges and ctenophores [53]. The resulting Maximum Likelihood (ML) tree (Figure 1) shows a strongly supported LIM-HD ingroup; note that we judge significance to be ≥ 70% bootstrap support for ML, Maximum Parsimony (MP), and Minimum Evolution (ME), and > 95% Bayesian posterior probability (Ba). The LIM-HD family is rooted between an Apterous-Arrowhead clade and a Tailup-Lim3-Lim1-Lmx clade, but these largest ingroup clades received significant or nearly significant support only by the ML method (72% and 67%, respectively). The tree includes two other nodes above the subfamily level: one uniting the Lim3, Lim1, and Lmx subfamilies, which received significant ML (75%) and Ba (98%) support, and one uniting the Lim3 and Lim1 subfamilies, which received significant ML (80%), MP (72%), and ME (74%) support, and nearly significant Ba (94%) support. The higher-level relationships among LIM-HD subfamilies shown in our tree are nearly identical to those shown in the LIM-HD phylogeny of Srivastava *et al*. [54]. The only difference is that their tree places the Tailup subfamily as sister to the Apterous-Arrowhead clade, instead of as sister to the Lim3-Lim1-Lmx clade. In our tree, the subfamilies themselves received robust support by all four methods, and the *Neanthes apterous*-related and *lim1*-related genes grouped within the Apterous and Lim1 subfamilies, respectively. Therefore, we firmly conclude that each is an ortholog of that subfamily, and we name these genes *Narap* and *Narlim1*.

### Expression of *Nar*-*ap*

**Figure 2:**
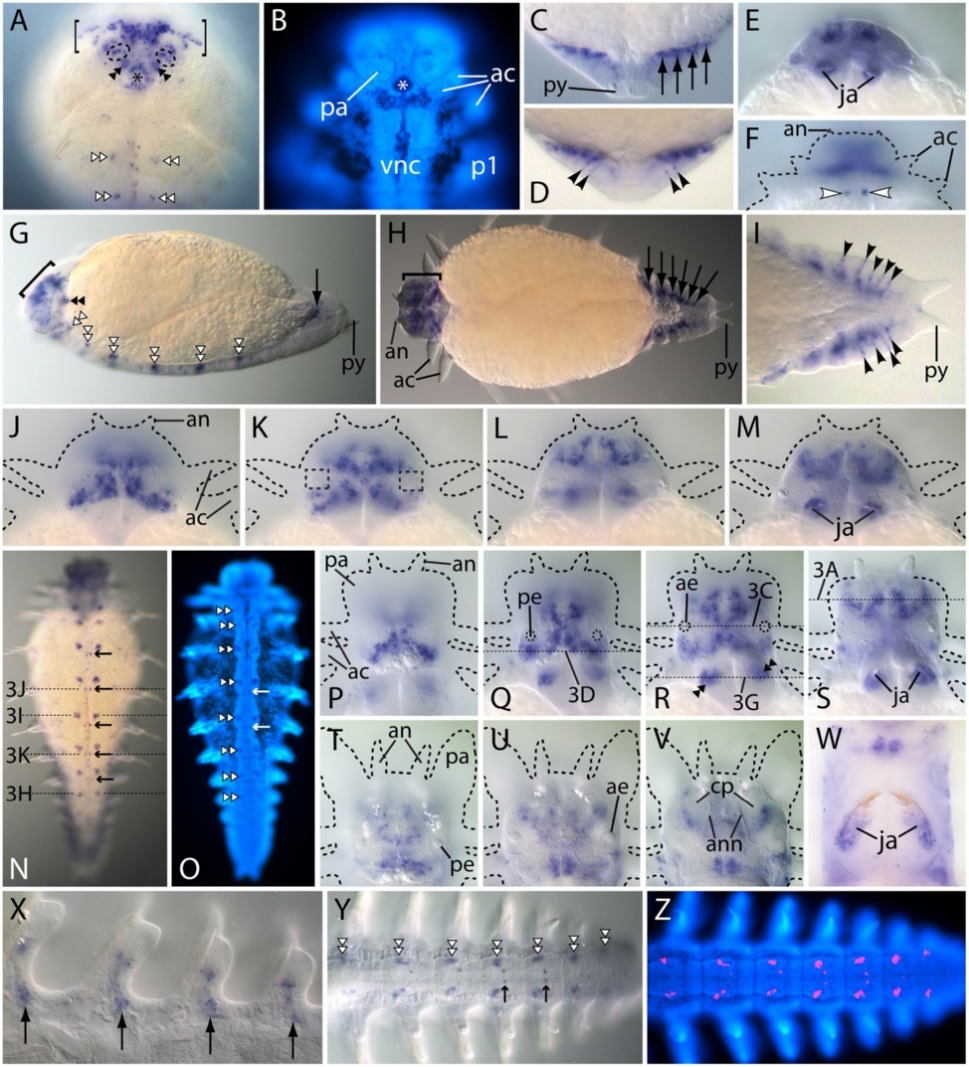
**Expression of *Nar*-*ap *during juvenile development. **(**A**), (**B**), (**N**), (**O**), (**Y**), (**Z**) Ventral views, anterior to the top (but to the left in (**Y**), (**Z**)). (**C**-**F**), (**H**-**M**), (**P**-**X**) Dorsal views, anterior to the top (but to the left in (**H**), (**I**), (**X**)). (**G**) Lateral view, anterior to the left. (**B**), (**O**), (**Z**) Hoechst 33342 fluorescence ((**B**) is a different specimen than that shown in (**A**); (**O**) and (**Z**) are the same specimen as that shown in (**N**) and (**Y**), respectively). (**J-M**), (**P-S**), (**T-V**) Sets of contiguous focal planes (dorsal to ventral) of the head, which is outlined with *dashes*. Expression domains in this figure are indicated by the following symbols: *arrowheads *(*black*) ectoderm of nascent segments, *arrowheads *(*white*) presumed tegumentary neurons, *arrows *(*large*) dorsal mesoderm, *arrows* (*small*) medial cell clusters of the nerve cords, *brackets* brain, *dashed ellipses *palp bases, *double arrowheads *(*black*) pharynx, *double arrowheads *(*white*) lateral cell clusters of the nerve cords. (**A-D**) Hatchling stage. (**A**), (**B**) Anterior end. An *asterisk* marks the foregut lumen. (**C**), (**D**) Posterior end (same specimen; (**C**) is a Nomarski image and (**D**) is a brightfield image). (**E**) Mid 3-cheatiger stage, anterior end. (**F**), (**G**) Late 3-chaetiger stage. (**F**) Anterior end, out of focus and outlined with *dashes*. (**H-M**) 4-chaetiger stage. The posterior end is shown in (**I**), and *dashed boxes *in (**K**) delimit regions of eye development. (**N**), (**O**) 7-chaetiger stage. (**P-S**) 8-chaetiger stage. (**T-W**) 13-chaetiger stage. (**X-Z**) 17-chaetiger stage. (**X**) Notopodia of the 4 posterior-most chaetigers. (**Z**) Expression is false-colored red. *ac *anterior cirri, *ae *anterior eye, *an *antenna, *ann *antennal nerve, *cp *corpus pedunculus, *ja *jaw, *p1 *1st parapodium, *pa *palp, *pe* posterior eye, *py *pygidium, *vnc *ventral nerve cords.

At the hatchling stage, *Nar*-*ap* mRNAs are distributed widely in the head (Figure 2A; compare with Figure 2B). In particular, transcripts occur in many areas of the brain (Figure 2A, brackets), in anterior cells of the palp bases (Figure 2A, dashed ellipses), and in cells encircling the foregut opening (Figure 2A, asterisk). A bilateral pair of cell clusters within the pharynx expresses *Nar*-*ap* (Figure 2A, black double arrowheads), and low-level *Nar*-*ap* activity occurs in segmentally iterated cell clusters within the nerve cord ganglia (Figure 2A, white double arrowheads). At the posterior end, *Nar*-*ap* expression is evident primarily in mesoderm (Figure 2C, arrows) and in several ectodermal cells (Figure 2D, arrowheads).

Inspection of mid 3-chaetiger juveniles reveals that the *Nar*-*ap* pharyngeal expression corresponds to cells surrounding the developing jaws (Figure 2E). The late 3-chaetiger stage is the only stage at which we observed *Nar*-*ap* expression in the achaetous ring; it occurred in two dorsal superficial cells (Figure 2F, white arrowheads), which are probably acetylated alpha-tubulin (AAT)-immunoreactive tegumentary neurons that connect to the cephalic nervous system (data not shown). (Note that antibodies to AAT are used to label neuronal cell processes (axons and dendrites) and cilia; see below). Also at this stage, expression in the nerve cord ganglia is more evident, being present in discrete cell clusters of every segment (including nascent, achaetigerous segments) except those in the approximately posterior one-third of the worm (Figure 2G, white double arrowheads).

By the 4-chaetiger stage, *Nar*-*ap*-positive mesoderm is present in at least six nascent segments posterior to the main yolk mass (Figure 2H, arrows), and the ectodermal expression (observed in five nascent segments) appears as laterally positioned stripes that vary in length (Figure 2I, staggered arrowheads). The brain’s posterodorsal cortex shows particularly strong *Nar*-*ap* expression that encompasses cells at the posterior prostomial border (Figure 2J). A slightly deeper focal plane shows a distinct absence of expression in mid-lateral prostomial regions that include the developing eyes (Figure 2K, dashed boxes). Most other portions of the prostomium within this focal plane (predominantly brain) are *Nar*-*ap*-positive (Figure 2K). Deeper focal planes exhibit expression in bilaterally paired U-shaped expression domains occupying the anterior half of the head (Figure 2L, M), and persistent expression occurs around the jaws (Figure 2M).

In 7-chaetiger juveniles, the ventral nerve cords (VNCs) exhibit prominent bilateral pairs of *Narap*-positive cell clusters that associate with eight pairs of ganglia, including the subesophageal ganglia (most anterior) (Figure 2N; Figure 2O, double arrowheads). In addition, a smaller bilateral pair of *Narap*-positive VNC cell clusters is evident posterior and medial to each of the larger cluster-pairs (Figure 2N and O, arrows). In the 8-chaetiger juvenile brain, *Narap* mRNAs continue to accumulate in the posterodorsal cortex (Figure 2P); this staining forms the bottom half of an X-like pattern, the top half of which is seen in the next two deeper focal planes (Figure 2Q, R). As was the case in 4-chaetiger juveniles, a large region anterior as well as medial to the eyes is devoid of *Narap* activity (Figure 2Q, R). A slightly deeper focal plane exposing an anteroventral portion of the brain (Figure 2S) shows persistent expression in the U-shaped patterns described above for 4-chaetiger juveniles. Starting in the medial brain, each ‘U’ wraps widely around the ‘antennal ganglion’ (*sensu*[48]) to a position between the antenna and palp at the prostomium’s anterior terminus. We also observed continued expression around the jaws (Figure 2R (double arrowheads), 2S).

**Figure 3:**
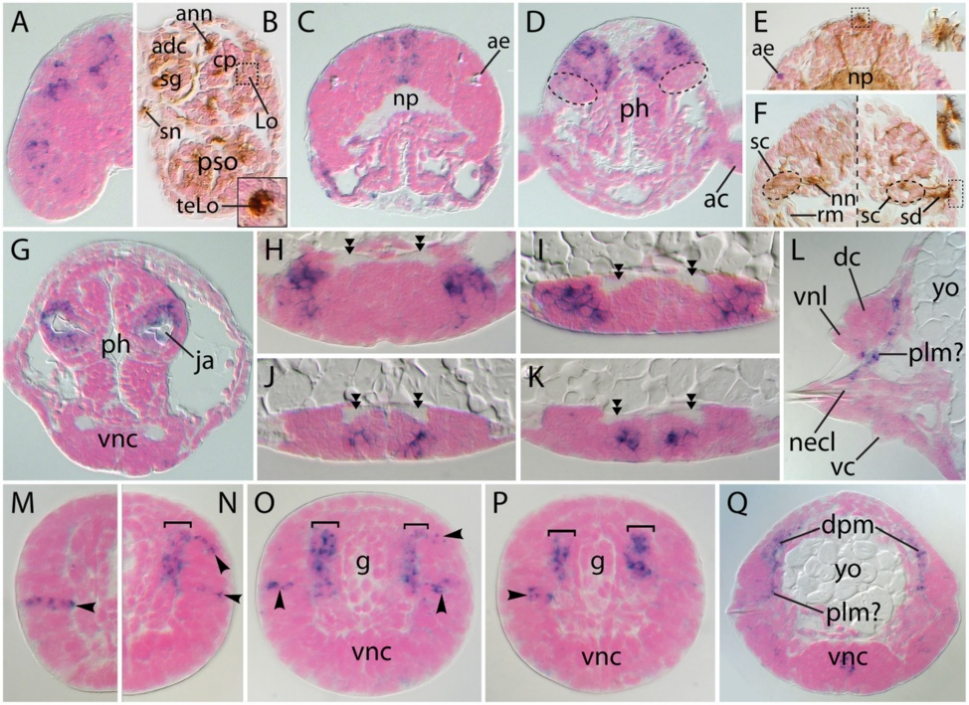
**Histologic cross sections showing further details of *Nar*-*ap *expression. **All sections are orthogonal to the anteroposterior (AP) axis; dorsal is to the top. (**A**-**G**) 8-chaetiger stage. (**H**-**Q**) 7-chaetiger stage. (**B**), (**E**), (**F**) Acetylated α-tubulin immunolabeling; cilia and neuronal cell processes are brown. (**A**), (**B**) Just posterior to the antennal ganglia, 10 μm anterior to the brain’s neuropil. Inset image in (**B**) is 10 μm anterior to, and a blowup of, the *Lo *region delimited with a *dashed box*. (**C**) Cephalic section at the level of the anterior eyes. (**D**) *Dashed ellipses *circumscribe areas expected to each contain a nuchal organ. (**E**) Same AP position as (**C**). The inset ciliary tuft is 5 μm anterior to, and a blowup of, the *boxed area*. (**F**) Same AP position as (**D**), showing nuchal organ locations in the posterior brain. The three parts of this panel - left, right, and inset ciliary tufts (a blowup of the *boxed area*) - are consecutive sections 5 μm apart (from anterior to posterior, respectively). (**G**) Pharyngeal section at the level of the jaws. (**H-K**) Ventral nerve cords. *Double arrowheads* point to longitudinal nerve tracts. (**L**) Posterior section through a sixth parapodium. (**M-Q**) Pre-chaetigerous segments near the posterior growth zone, arranged from youngest to more mature and showing ectodermal (*arrowheads*) and mesodermal (*brackets*) expression domains. *adc* anterodorsal cortex of the brain, *ae *anterior eye, *ann* antennal nerve, *cp *corpus pedunculus, *dc *dorsal cirrus, *g* gut, *Lo *Langdon’s organ, *necl *neuropodial chaetal lobe, *nn *nuchal nerve, *np *neuropil, *plm*? presumed parapodial levator muscle, *pso *palp sense organs, *rm *nuchal organ retractor muscle, *sc *nuchal organ sensory cells, *sd* nuchal organ sensory dendrites, *sg*/*n *stomatogastric ganglion/nerve, *teLo *terminal endings of sensory-cell peripheral processes from Langdon's organ, *vc *ventral cirrus, *vnc *ventral nerve cords, *vnl *ventral notopodial ligule, *yo *yolk.

Figure 3 presents additional details of *Narap* expression in 7- and 8-chaetiger juveniles. Comparison of a transverse section halfway through a prostomial U-shaped expression domain (Figure 3A; see dashed line in Figure 2S) with an equivalent section from an AAT-labeled specimen (Figure 3B) reveals that the medial portion of a ‘U’ likely consists of cells in the brain’s anterodorsal cortex and, ventral to this, in a stomatogastric ganglion (see [48]). The lateral portion of each ‘U’ likely corresponds to a Langdon’s organ (Figure 3B) (see [55,56]). Note that we deduced this organ’s identity from the relative positions of the neighboring and more distinctive antennal nerve and corpus pedunculus, and from its own telltale sensory cilia (Figure 3B, inset; see also [48]). Expression at this transverse level of the head also occurs in the palp’s lateral portion, possibly in sensory organs (Figures 3A, B).

In a section through the anterior eyes (Figure 3C; see dashed line in Figure 2R), *Nar*-*ap*-positive cells occupy a medial domain dorsal to the neuropil. A comparable section from an AAT-labeled specimen (Figure 3E) reveals a previously unknown dorsal ciliated organ, possibly connected to the neuropil via a bilateral pair of oblique nerves. Description of this organ is beyond the scope of the current work, but coincident *Nar*-*ap* expression (Figure 3C) suggests a role for this gene in its development.

In a section taken just posterior to the posterior eyes (Figure 3D; see dashed line in Figure 2Q), *Narap* expression appears limited to the dorsolateral and ventromedial sides of the brain. Comparable sections of an AAT-labeled specimen (Figure 3F) reveal that the nuchal organs (chemosensory organs comprised of a nuchal nerve, a cluster of sensory cells, their dendrites, external ciliation, and internal retractor muscles; see, for example, [48,57]) occupy ventrolateral positions in this portion of the brain. The lack of *Narap* expression here (Figure 3D, dashed ellipses) suggests minimal *Narap* function in nuchal organ differentiation. This result goes against our above prediction (see Background) of *ap* expression in *Neanthes* olfactory organs.

A transverse section through the jaws indicates that pharyngeal expression of *Nar*-*ap* is limited to the epithelial sac surrounding each jaw (Figure 3G). In regard to the *Nar*-*ap*-expressing VNC cell clusters, transverse cross sections through each cluster type (see dashed lines in Figure 2N) reveal that, first, the larger anterior type, in both a nascent segment (Figure 3H) and a more mature segment (Figure 3I), includes no fewer than ten cells (accounting for cluster thickness along the anteroposterior axis) and occupies much of the lateral portion of each ganglion. Second, the smaller clusters reside medially and at a median or basal level in each ganglion, and consist of no fewer than three cells (Figures 3J, K).

Transverse sections through the posterior portion of a 7-chaetiger juvenile clarify spatial details of *Narap* expression in nascent segments and outgrowing parapodia (Figures 3L-Q). First, ectodermal expression, observed only prior to parapodial outgrowth, exists in narrow rows that are one to two cells in height, vary in length, and number two per hemisegment, with one residing at a dorsal level and the other residing at a roughly median level (Figures 3M-P, arrowheads; multiple sections are presented to show variation in the length of these rows, and that their positions do not always overlap - with each other or with *Narap*-positive mesoderm). Second, mesodermal expression occurs in thick dorsolateral cell blocks that are delimited roughly by the positions of the *Narap*-positive ectodermal rows (Figures 3N-P). In a slightly older segment (Figure 3Q), the position of *Narap*-positive mesodermal cells corresponds to the dorsal parapodial muscles [48,52]. Moreover, the narrow band of *Narap*-positive mesoderm extending ventrolaterally from the vicinity of the dorsal parapodial muscles to the neuropodial chaetal lobe likely corresponds to the developing parapodial levator muscle (Figures 3L, Q; see also [48,52]).

At the 13-chaetiger stage, *Narap* activity persists in the brain’s posterodorsal cortex (Figure 2T), and appears to be continuous with posteroventral brain expression (Figures 2U, V), but expression posterior to and between the eyes has become less intense (Figures 2T, U). The anterior U-shaped domains continue expressing *Narap* in the brain’s anterodorsal cortex (Figure 2U). Ventrally, however, expression in the medial portion of each ‘U’ (that is, expression in the presumed stomatogastric ganglia) appears to have largely ceased, but the lateral expression persists (Figure 2V). This staining wraps around the lateral sides of each corpus pedunculus, localizing to the expected positions of the Langdon’s organs (see Figure 5E of [48]), corroborating our above presumption based on AAT labeling and sections of the 8-chaetiger stage prostomium. In the jaw sacs, *Narap*-expressing cells are detected only in specimens having undergone longer color reactions (Figure 2W), indicating attenuation of expression (note that negative control experiments run with sense probes for the same amount of time showed no equivalent staining; also, the *Narap*-positive cell clusters near the top of Figure 2W reside in the posteroventral brain).

In accord with the pattern observed in 7-chaetiger juveniles, outgrowing parapodia of 17-chaetiger juveniles also express *Nar*-*ap* in what appears to be a dorsal sub-ectodermal domain, but only within the posterior portion of the segment (Figure 2X). This indicates that the anterior set of dorsal parapodial muscles develop in the absence of *Nar*-*ap* activity. *Nar*-*ap* expression patterns in the VNCs of 17-chaetiger juvenile posterior segments (Figure 2Y, Z) are identical to those observed at earlier stages, indicating that serially homologous *Nar*-*ap*-positive VNC cell clusters develop in each newly added segment.

### Expression of *Nar*-*lim1*

**Figure 4:**
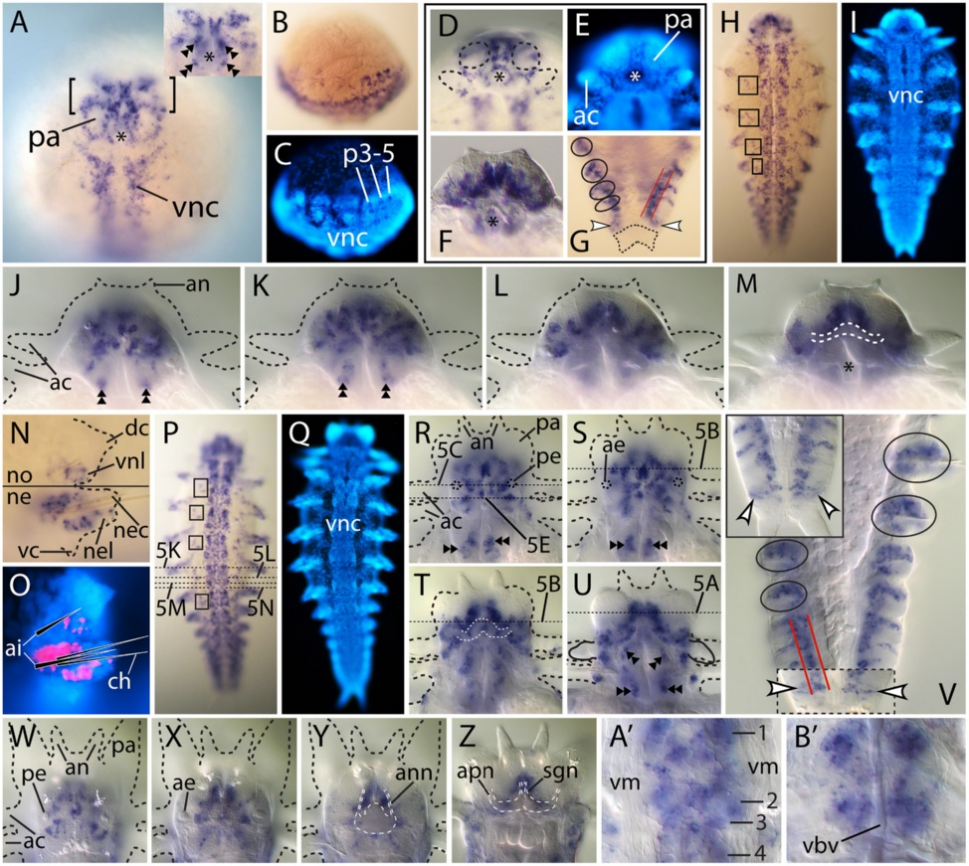
**Expression of *Nar*-*lim1 *during juvenile development. **(**A**), (**D**), (**E**), (**G**), (**H**), (**I**), (**P**), (**Q**), (**U**), (**V**), (**Z**-**B**’) Ventral views, anterior at top. (**B**), (**C**) Ventrolateral views, anterior at left. (**F**), (**J**-**M**), (**R**-**T**), (**W**-**Y**) Dorsal views, anterior at top. (**N**), (**O**) Anterolateral parapodial views, distal at right. (**A**), (**D-F**), (**M**) An *asterisk *marks the foregut. (**C**), (**E**), (**I**), (**O**), (**Q**) Hoechst fluorescence of the preceding specimen. (**J-M**), (**R-T**), (**W-Y**) Contiguous cephalic focal planes (dorsal to ventral). (**M**), (**T**), (**Y**), (**Z**) *White dashes* outline visible neuropil and cephalic nerves. Expression domains are indicated by: *arrowheads *presumed posterior growth zone, *boxes *presumed peripheral neurons, *brackets *brain, *double arrowheads *pharynx, *ellipses *parapodia, *parallel red lines *nascent segmental mesoderm. (**A-C**) Hatchlings. (**A**) The inset image shows a deeper pharyngeal focal plane. (**D-G**) Mid 3-chaetiger stage. (**D-F**) Anterior end. (**D**) Cephalic appendages are outlined. (**F**) Mid focal plane of the brain. (**G**) Posterior end, with pygidium outlined. (**H-O**) Late 4-chaetiger stage. (**N**), (**O**) Third parapodium. (**O**) False-colored expression and schematic bristles. (**P-U**) 7-chaetiger stage. (**V**) 7-chaetiger juvenile (inset image) and 9-chaetiger juvenile (main image) posterior end. The *boxed region *is from a shallower focal plane. (W-Z) 13-chaetiger stage. (**A’**), (**B’**) 20-chaetiger stage. Contiguous focal planes of VNC ganglia in a mid-body segment ((**A’**) is at a median focal level, and (**B’**) is deeper). *Numbered lines *in (**A’**) indicate the root locations of segmental nerves 1 to 4 in the right hemisegment. *ac *anterior cirrus, *ae *anterior eye, ai *acicula*, *an *antenna, *ann *antennal nerve, *apn *axial palp nerve, *dc *dorsal cirrus, *ne *neuropodium, *nec *neuropodial chaetal lobe, *nel *neuropodial ligule, *no* notopodium, *p3*-*5 *third to fifth parapodia, *pa* palp, *pe *posterior eye, *sgn *stomatogastric nerve, *vc* ventral cirrus, *vbv *ventral blood vessel, *vm *ventral longitudinal muscle, *vnc *ventral nerve cords, *vnl *ventral notopodial ligule.

In hatchlings, the head is a dominant territory of *Nar*-*lim1* activity; its transcripts are detected around the foregut opening (Figure 4A, asterisk), in cells circumscribing the palps, and within the brain (Figure 4A, brackets). Within the pharynx, two pairs of bilateral cell clusters, one dorsal and one ventral, are *Nar*-*lim1*-positive (Figure 4A, double arrowheads). Within the VNCs, *Nar*-*lim1* transcription extends beyond regions expressing *Nar*-*ap* to the extent that *Nar*-*lim1* staining reflects the basic nerve-cord structure (Figure 4A; compare with Figure 2A). At the hatchling posterior end, nascent segments show intense *Nar*-*lim1* signal in preformed parapodia (Figures 4B, C).

At the mid 3-chaetiger stage, a superficial ventral view of the head (Figures 4D, E) reveals persistent *Narlim1* expression around the foregut opening and between the palps. Other *Narlim1*-positive cells reside in the furrows between the outgrowing palps and anterior cirri, and at the posterior bases of these cirri. A deeper focal plane (Figure 4F) captures intensely staining, bilaterally paired expression domains in the medial brain that roughly parallel the superficial/ventral columns of *Narlim1*-positive cells between the palps. Brain regions posterolateral to these medial domains also exhibit considerable *Narlim1* expression (Figure 4F). Parapodial expression at this stage (Figure 4G, ellipses) can be discerned in ectoderm (lateral-most staining) and mesoderm (Figure 4G, between parallel red lines). Expression immediately anterior to the pygidium (Figure 4G, arrowheads; pygidium outlined) does not appear to be in register with the segmentally repeated parapodial staining, and hence may be associated with the posterior growth zone (PGZ). However, to rule out that this expression reflects an earlier stage of parapodial development, it would need to be mapped relative to the expression of genes that mark the PGZ (for example, *caudal*, *evenskipped*) and/or newly added segments (for example, *hedgehog*, *wingless*, *engrailed*, *NK* genes) [7,58-60].

In late 4-chaetiger juveniles, *Narlim1* transcripts accumulate in every VNC ganglion (Figure 4H; compare with Figure 4I). A segmentally repeated expression pattern lateral to the nerve cords and posterior to the parapodia is also evident (Figure 4H, boxes). While further investigation is required to determine the identities of these *Narlim1*-positive cells, we provisionally assign them to the segmental peripheral nervous system (see [48,61]). In the brain, clusters of *Narlim1*-positive cells populate the posterodorsal cortex, but in a manner lacking a strong bilateral pattern (Figure 4J). At a slightly deeper focal plane (Figure 4K), *Narlim1* expression is widespread in the brain’s anterodorsal cortex, occurring in numerous cell clusters with a somewhat stronger bilateral pattern. Expression in the anteromedian brain cortex (Figure 4L) changed little from that described above for mid 3-chaetiger juveniles, and it appears to be more or less continuous with expression in the brain’s anteroventral cortex (Figure 4M): at both stages, and in both the anteromedian and anteroventral brain regions, staining occurs in the lateral-most ganglia and in a large pair of ganglia abutting the midline anterior to the neuropil (Figures 4L, M; compare with Figure 4F). We also observed continued pharyngeal expression (double arrowheads in Figures 4J, K). Parapodia at the late 4-chaetiger stage have undergone modest outgrowth, and their *Narlim1* transcripts associate with the developing ventral notopodial ligule, the chaetal bases, and the neuropodial ligule (Figures 4N, O).

**Figure 5:**
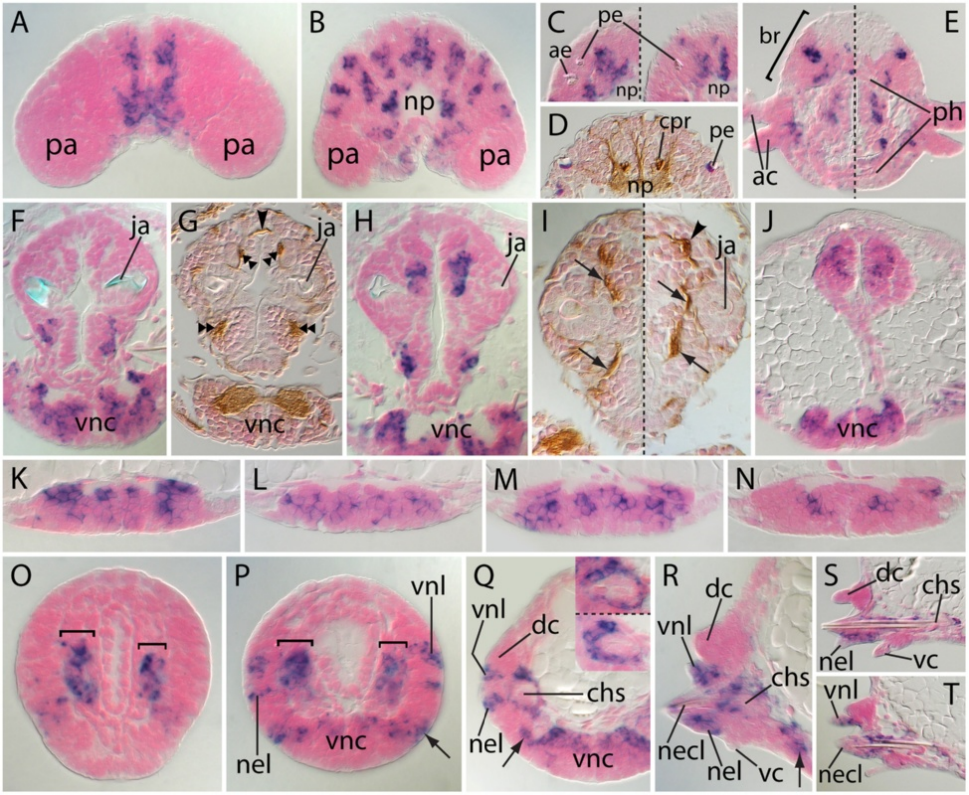
**Histologic cross sections of 7-chaetiger juveniles showing further details of *Nar*-*lim1 *expression. **All sections are orthogonal to the AP axis; dorsal is to the top. *Dashed lines *separate sections spaced apart by 5 μm in (**C**), (**E**), (**Q**) and 10 μm in (**I**) (the more posterior section is to the right or below the line). (**D**), (**G**), (**I**) Acetylated α-tubulin immunolabeling. (**A**) Same AP position as Figures 3A, B. (**B**) Anterior-most level of the brain’s neuropil. (**C**) Upper quadrant of the head; the mid-sagittal plane is flush with each image’s right edge. (**D**) Same AP position as (**C**). (**E**) Posterior brain and anterior pharynx. (**F**-**J**) Three positions within the pharynx: anterior jaws (**F**), (**G**), posterior jaws (**H**), (**I**), and caudal-most (**J**). *Arrowheads *point to exposed portions of commissural nerves; *double arrowheads *point to longitudinal nerves; *arrows *point to exposed portions of medial nerves connecting ipsilateral ganglia. (**K**-**N**) Various positions within the 5th and 6th ganglion pairs of the ventral nerve cords. (**O**-**T**) *Nar*-*lim1 *expression during parapodial development. *Brackets *indicate mesodermal expression; *arrows *mark expression in parapodial ganglia. (**O**) Nascent segment nearest the pygidium. (**P**) Nascent segment approximately 20 μm anterior to (**O**). (**Q**) Youngest outgrowing parapodium; approximately 50 μm anterior to (**P**). Inset images show additional sections through the chaetal sac, 10 and 15 μm anterior to main image, respectively. (**R**) Youngest chaetigerous (sixth) parapodium. (**S**), (**T**) First pair of parapodia (left and right, respectively) from the same section; one shows the *nel *(**S**), and the other the *vnl* (**T**). *ac *anterior cirri, *ae *anterior eye, *br* brain, *chs *neuropodial chaetal sac, *cpr *ciliary photoreceptor cells, *dc *dorsal cirrus, *ja *jaw, *necl *neuropodial chaetal lobe, *nel *neuropodial ligule, *np *neuropil, *pa* palp, *pe *posterior eye, *ph *pharynx, *vc *ventral cirrus, *vnc *ventral nerve cords, *vnl *ventral notopodial ligule.

At the 7-chaetiger stage, ongoing *Narlim1* activity occurs in the VNCs (Figure 4P; compare with Figure 4Q), presumed segmental peripheral nervous system (Figure 4P, boxes), and pharynx (Figures 4R-U, double arrowheads). Expression in the head is complex (Figures 4R-U), but despite this, staining in anteroventral head regions (Figures 4T, U) resembles previously described stages. The aforementioned parapodial and presumed PGZ expression patterns are also evident in 7- and 9-chaetiger juveniles (Figure 4V). Analysis of transversely sectioned specimens helps to clarify details of *Narlim1* expression at this stage (Figure 5). A section through the anterior prostomium (Figure 5A; see dashed line in Figure 4U) shows narrow strips of *Narlim1*-positive cells linking two other *Narlim1*-positive domains: the ganglia anterior to the neuropil, and the tissue between the palp bases. A section through the anterior neuropil (Figure 5B; see dashed line in Figure 4S, T) reveals *Narlim1* activity in the epithelium between the palp bases, and in no fewer than eight bilateral cell-cluster pairs within the brain. In the ocular region (Figure 5C; see dashed line in Figure 4R), *Narlim1*-positive domains medial to the posterior eyes appear to co-localize with internal brain structures exhibiting intense AAT labeling (Figure 5D). These are in all likelihood the ciliary photoreceptor cells [62]. A posterior transverse section of the head (Figure 5E; see dashed line in Figure 4R) documents *Narlim*-positive cell clusters in dorsolateral and ventral sectors of the brain that potentially overlap with *Narap*-positive cells and the nuchal organs, respectively. Other expression at this transverse level is present in the achaetous ring; it occurs just medial to the anterior cirri, presumably in cirral ganglia (Figure 5E).

In transverse sections of the 7-chaetiger juvenile pharynx, we observed *Narlim1* activity in bilaterally paired, ventrolateral cell groups just below the anterior jaws (Figure 5F). Comparable AAT-labeled sections reveal a ventral longitudinal nerve adjacent to each cell group (Figure 5G). In addition, *Narlim1*-positive cells neighbor dorsal longitudinal nerves in sectors that are above and medial to the jaws (compare Figures 5G and H). Because *Narlim1*-positive pharyngeal cells associate with neural elements (for example, longitudinal nerves, dorsoventral nerves connecting ipsilateral cell groups, and commissural nerves connecting contralateral cell groups) (Figures 5G, I), we conclude that they are integrated into pharyngeal ganglia. *Narlim1* expression is also evident posterior to the jaws (Figures 5E, J), in ganglia that also express *dachshund* and *optomotor blind*[8].

Transverse sections through the fifth and sixth ganglion pairs clarify the segmental expression pattern of *Narlim1* within the 7-chaetiger-stage VNCs (see dashed lines in Figure 4P). In a mid-segmental registry, near the second segmental nerve roots (see [40,61]), VNC expression is most intense within dorsolateral sectors of the ganglia (Figure 5K). Posterior in the segment, near the fourth segmental nerve roots, light *Narlim1* expression occurs at a median level across the VNC (Figure 5L). In the anterior of the adjacent segment, near the first segmental nerve roots, expression is similar to that seen in Figure 5K, but with basal extension of lateral expression (Figure 5M). In VNC regions recognized by apparent gaps in expression, that is, between the first and second segmental nerves, expression is primarily restricted to a bilateral pair of cell clusters lying near the midline at a median level within the cords (Figure 5N).

Analysis of transverse sections through the newly added posterior segments of 7-chaetiger juveniles enabled us to precisely identify the developing parapodial structures that express *Narlim1*. Transcripts first occur in thick blocks of cells in nascent ventral mesoderm (Figure 5O and P, brackets). As parapodial outgrowth proceeds, these cells come to surround the neuropodial chaetal sac (Figures 5Q-S), forming the neuropodial chaetal sac muscles. The notopodial chaetal sac develops later than that of the neuropodium, and no evidence of *Narlim1* expression in its musculature was observed. Prior to outgrowth, ectodermal *Narlim1* expression is evident in three distinct domains (Figure 5P); two align roughly with the top and bottom of the mesodermal expression domain, and one lies just lateral to the nerve cord. The identities of these domains become clear as outgrowth proceeds. The expression domain adjacent to the nerve cord forms the medial cells of the parapodial ganglion, and the successively more dorsal domains form the anlagen of the neuropodial ligule and the ventral notopodial ligule (Figures 5Q-S). Morphogenesis of the dorsal notopodial ligule occurs during later stages of parapodial outgrowth [48] and was not observed in this study.

In 13-chaetiger juveniles, the posterior brain bears two separate *Narlim1*-positive cell clusters posterior to each posterior eye, and a pair of clusters on the midline between these eyes (Figure 4W). A deeper focal plane (Figure 4X) shows a *Narlim1*-positive cluster medial to each anterior eye. How these patterns relate specifically to similar patterns in earlier stages is difficult to judge. However, one pattern remains comparatively static among the stages: medial expression in the anterior brain. This occurs in dorsal, median, and ventral levels of the cortex (Figures 4X, Y, Z, respectively), where the median and ventral expression together corresponds to a pair of stomatogastric ganglia (of the first pair of stomatogastric nerves) [48]. We observed continued pharyngeal and VNC expression at this stage (data not shown), as well as in 20-chaetiger-juvenile VNC ganglia (Figures 4A’, B’), which exhibit *Narlim1* activity that is largely consistent with the VNC patterns described for earlier stages, suggesting persistent transcription in the same cell types.

## Discussion

### *Apterous*, *lim1*, and appendage *evo*-*devo*

We previously assayed the expression of *Neanthes Distalless*, *dachshund*, and *optomotor blind* to investigate whether conserved mechanisms of appendage development exist across the Bilateria [8]. Expression of these genes does not map to the dorsoventral and proximodistal fields that might be predicted by comparison to arthropod and vertebrate appendages, implying independent evolution of annelid appendage development. Here, we supplement our previous work by discussing whether parapodial expression of *Narap* and *Narlim1* supports conservation of limb developmental between bilaterian phyla. As outlined in the Background section, extensive *Narap* expression in developing notopodia would support homology of limb organization into dorsal and ventral elements in annelids and arthropods. However, the two narrow rows of *Narap*-positive cells in parapodial ectoderm (arrowheads in Figure 2I and Figures 3M-P) cease their expression prior to outgrowth, and notopodial *Narap* expression during outgrowth is limited to muscles. Therefore, *Narap* activity is not similar in its broad scope to the activity of *ap* homologs in the dorsal division of the arthropod limb; hence this comparison yields little support for a branched appendage architecture in the common ancestor of lophotrochozoans and ecdysozoans. In addition, parapodial *Narap* expression bears minimal resemblance to the limb activities of vertebrate *ap* orthologs, *Lhx2* and *Lhx9*, which function in the progress zone (distal mesoderm) to integrate outgrowth with anteroposterior and proximodistal patterning [63,64]. The vertebrate *lim1* orthologs, *Lhx1* and *Lhx5*, play no role in limb development [64], but *lim1* in *Drosophila* and *Tribolium* is expressed in multiple developing leg segments, where it is required for proper proximodistal patterning [20-22,25]. *Narlim1* expression in parapodia does not appear similarly organized. Rather, it may play roles in the morphogenesis of ligules, and in the development of a specific set of muscles. These contrasting patterns of *ap* and *lim1* expression in the developing appendages of *Neanthes* versus other bilaterians strengthen the conclusion of Winchell *et al*. [8] that pattern formation in parapodia evolved independently of that found in arthropod or vertebrate limbs. This suggests that Lhx genes were separately co-opted in these lineages from their apparent symplesiomorphic roles in neural differentiation to autapomorphic roles governing distinctly different aspects of limb outgrowth.

The absence of intricate morphologic and developmental-genetic similarities between annelid and arthropod appendages strongly suggests that these structures arose separately within the protostomes. This is further supported by molecular-phylogenetic findings, as recently published topologies place taxa lacking limbs comparable to parapodia and arthropodia basally within the Lophotrochozoa and Ecdysozoa, respectively (see [8]). While *de novo* evolution of parapodia and of arthropodia seems likely, two alternative hypotheses merit consideration. The first, predicated on the notion that evolutionarily related structures in disparate phyla may exhibit conflicting patterns of gene expression and morphology due to differential persistence and modification of ancestral conditions [65,66], posits independent divergence of parapodia and arthropodia from a common lateral structure in primitive protostomes. This hypothetical ancestral structure may have served a respiratory role, but was more likely sensory, given the developmental-genetic similarities and functional associations between sensory organs and appendages in modern bilaterians [65,66]. A second alternative to *de novo* evolution is the possibility that parapodia and arthropodia are transformational homologs of pre-existing structures in the separate ancestors of annelids and arthropods. Under this scenario, there is a distinct possibility that the pre-existing structures are still present in modern annelids and arthropods. The precedent for this consideration is the extensive developmental-genetic and morphologic similarity shared between outgrowing chondrichthyan gill arches and vertebrate fin/limb endoskeletons, which suggests that vertebrate paired appendages are transformational homologs of the earlier-evolved gill arches [67]. Paired fins/limbs likely originated in the Silurian (in the now-extinct osteostracan fishes), well after the origin of vertebrates, and their evolution can be further resolved as a process involving co-option of developmental mechanisms from the AP axis [9] and unpaired median fins [68]. Additional insights into the transformational and co-optive processes that spurred the evolution of vertebrate paired appendages may be gleaned from developmental-genetic comparisons between gnathostomes and their closely related extant outgroups, namely cyclostome fishes and cephalochordates. Unfortunately, no such clarity in modeling appendage evolution from pre-existing structures is immediately available on the protostome side of the tree. Panarthropod appendages (for example, lobopodia and arthropodia), as well as parapodia-bearing polychaetes [69], were already present in the Lower Cambrian, and recent molecular clock estimates date the origin of lophotrochozoans and ecdysozoans to the Lower Ediacaran [70]. The scarcity of fossils from this period makes it difficult to characterize the morphology of ancestral forms of Lophotrochozoa and Ecdysozoa. In addition, bodyplan disparity and lingering uncertainty in the deep phylogenetic relationships within these taxa complicate outgroup comparison.

### *Apterous*, *lim1*, and specification of muscle precursors

In the mesoderm of nascent segments, *Narap* and *Narlim1* are each expressed in morphologically undifferentiated and presumably separate groups of cells. Differentiation of these cells over the course of a few segment-additions results in the formation of distinct sets of parapodial muscles. The presumed parapodial levator muscle and posterior members of the dorsal parapodial muscles develop from *Narap*-positive mesoderm, while neuropodial chaetal sac muscles develop from *Narlim1*-positive mesoderm. These complementary expression patterns are reminiscent of the complementary/combinatorial expression of Lhx genes - that is, the ‘LIM code’ - that controls the differentiation of interneuron and motor neuron subsets in bilaterian nerve cords (for example, [71]). It is, therefore, tempting to speculate that parapodial myoblast diversification requires similar use of a LIM code. Perhaps other Lhx genes are also expressed separately or combinatorially in parapodial muscle precursors.

To our knowledge, this is the first report implicating a *lim1* gene in the development of a specific muscle type. *apterous*, however, is a known ‘muscle identity gene’ in *Drosophila*: it specifies precursors of six larval abdominal muscles and the direct flight muscles, and it non-cell-autonomously regulates early differentiation of the indirect flight muscles [72,73]. Other *ap* orthologs may have similar functions. For example, *Caenorhabditis ttx3* is expressed throughout embryonic development in a specific set of head muscles [74], and Apterous protein has been detected in limb muscles of the brine shrimp *Artemia franciscana*[12]. The activity of *ap* orthologs in disparate muscles of *Neanthes*, *Drosophila*, *Caenorhabditis*, and *Artemia* suggests that these genes were independently co-opted for roles in muscle development. Alternatively, their deployment might reflect conservation of a genetic network underlying myoblast diversification in protostomes. If true, this network may also involve NK homeobox genes, which show complementary expression in muscle precursors of *Drosophila* and of *Platynereis dumerilii* (a nereidid polychaete) [60].

### *Nar*-*ap* and *Nar*-*lim1* expression in the nervous system

Precise identification of the neural cells expressing *Narap* and/or *Narlim1* awaits thorough characterization of the developing nereidid nervous system, colocalization experiments with other neural markers, and the establishment of a genetic labeling strategy (for example, reporter-based tracing) in annelids that simultaneously reveals neuronal perikarya and projections (see [75]). However, the functions of bilaterian Lhx genes are generally conserved in central nervous systems (see Background), and remarkable developmental-genetic parallels have been demonstrated between the nervous systems of *Platynereis* and other bilaterians (for example, [76-78]). Thus, known neural functions of *ap* and *lim1* orthologs in model bilaterians serve as a sound platform for postulating the functions of these genes in the *Neanthes* nervous system.

#### Nar-ap

Certain post-mitotic interneurons in *Drosophila* and *Caenorhabditis* require *ap* for proper axonal pathfinding [33,74]. Moreover, *ap* is required in the *Drosophila* VNC connectives and brain to generate fascicules that contain only axons of *ap*-expressing interneurons [32,33]. Similarly, in vertebrates, differentiation of a set of post-mitotic spinal cord interneurons requires *Lhx2*/*9* for axonal pathfinding and fasciculation into ‘*Lhx2*/*9*-only’ bundles [36,37,40]. These findings suggest that *Narap* functions in axonal path finding and fasciculation of brain and VNC interneurons.

*Drosophila ap* has a second role in post-mitotic interneurons: controlling neuropeptide production. In one interneuron subset of each VNC ganglion, *ap* regulates FMRF-amide synthesis, and in two other subsets, it regulates expression of peptide biosynthetic enzymes leading to as yet unknown neuropeptides [34,79]. In the *Drosophila* brain, *ap* is required for the production of leucokinin neuropeptides [35], and certain *ap*-expressing interneurons innervate the neurosecretory ring gland [33]. These observations suggest that *Narap* might play a role in neuropeptide synthesis. Consistent with this, the posterior brain of *Neanthes* shows intense *ap* expression through the 17-chaetiger stage (the latest stage analyzed; data not shown). Ganglia in this portion of the nereidid brain contain concentrations of neurosecretory cells that show enhanced activity during caudal regeneration and/or later life history stages; they therefore appear to function as hormone-releasing centers, controlling growth and sexual maturation (for example, [80-83]).

*Lhx2* functionality in developing vertebrate brains indicates that neural activity of *ap* orthologs is not limited to post-mitotic neurons. In mouse, for example, *Lhx2* plays critical roles in forebrain neurogenesis by specifying fates of the neocortical and hippocampal precursor cells, and by regulating tissue patterning of the pituitary gland [84-86]. Supporting a neurogenesis role for *ap* in nereidid polychaetes, *ap* expression in the developing larval brain of *Platynereis* overlaps significantly with the expression of numerous other genes whose vertebrate orthologs are known to regulate early morphogenesis of the telencephalon [77].

Rincón-Limas *et al*. [41] inferred conserved expression of *ap* orthologs in olfactory organs across the Bilateria based on *Lhx2* expression in epithelia surrounding the embryonic nasal pits of mice and *ap* expression in the antennae and maxillary palps of flies. In further support of this argument, the differentiation of mouse olfactory neurons requires *Lhx2*[40], and squid *ap* mRNAs are present in developing olfactory organs and olfactory lobes of the brain [87]. Polychaete nuchal organs are putatively olfactory, reside partially within the brain, and bear ultrastructural likeness to vertebrate olfactory epithelia [57,88]. However, we did not detect *Narap* expression in the developing nuchal organs of 8-chaetiger juveniles, and we suspect that *Narap* mRNAs are also absent from younger juveniles’ nascent nuchal organs. Although this casts doubt on the hypothetically conserved role of *ap* orthologs in olfactory differentiation, the identities of *Narap*-positive cells in other potentially chemosensory organs, that is, the palps and Langdon’s organs, remain to be elucidated.

*ap* orthologs are also active in developing visual systems. The eyes and optic brain lobes of a squid express *ap*[87], as do the optic lobes of the *Drosophila* brain [41]. In vertebrates, *Lhx2* is required for eye morphogenesis [84,89], while both *Lhx2* and *Lhx9* are active in the optic tectum/superior colliculus (visual processing centers in the brain) [38,89]. The eyes of *Neanthes* clearly do not express *ap*; however, a role in visual system development cannot be rejected, as *Narap*-positive cells are observed between the posterior eyes (Figures 2Q, T), near the presumed location of the brain’s central optic neuropil [83].

#### Nar-lim1

The overall pattern of *Narlim1* expression in the VNC likely encompasses both motor neurons and interneurons, as these cell types show nerve cord expression of *lim1* orthologs in *Caenorhabditis*[90], *Drosophila*[43], and vertebrates (for example, [36,44]) (note that for *Drosophila* and vertebrates, *lim1*-expressing motor neurons target dorsal musculature). In these model bilaterians, *ap* and *lim1* orthologs are not co-expressed in nerve cord neurons; our *Neanthes* data are consistent with this pattern, but verification requires double-labeling experiments. Although the precise function of *lim1* in the *Drosophila* VNC is not known, loss of function and ectopic expression of *lim1* orthologs in *Caenorhabditis* and vertebrates result in axonal pathfinding and fasciculation defects in nerve cord neurons [36,44,90], indicating similarity of function to *ap*. The sustained expression of *Narlim1* during *Neanthes* VNC development suggests that it controls similar aspects in the final stages of neuronal differentiation.

We observed two general themes of *Narlim1* expression in the developing juvenile brain. The first is early and persistent expression, particularly in the medial portion of the anterior brain. Here, *Narlim1*-positive cells encompass most of the brain’s depth, are traceable from hatchlings through the 13-chaetiger stage (Figures 4A, F, K-M, R-T, X-Z), and include a prominent stomatogastric center. Such an early, persistent, and broad expression domain suggests a regulatory role for *Narlim1* in the initial specification and continued morphogenesis of this brain region. Detailed description of *lim1* expression and function is lacking for the *Drosophila* brain, but expression of vertebrate *lim1* orthologs is also suggestive of roles in the initial specification and morphogenesis of particular brain regions. In lamprey, for example, expression of the single *lim1* ortholog contributes to early division of the embryonic forebrain and hindbrain into neuromeric segments [91]. In addition, expression of *Lhx1* and *Lhx5* in embryonic brains of chicken and mouse, respectively, suggests that these genes play roles in controlling early forebrain formation and regionalization [92,93]. Consistent with this, functional analysis of *Lhx5* in zebrafish embryos demonstrates a role for this gene in promoting forebrain formation by inhibiting the caudalizing effect of Wnt signaling [94].

The second general theme we observed is continuous expression in posterodorsal portions of the brain, but in patterns that are difficult to trace between stages (compare Figures 4J, R, W). Such patterns may be the result of dynamic spatiotemporal expression among relatively stationary sets of cells, or of movement of *Narlim1*-expressing cells. We suspect the latter contributes most significantly to these patterns, as *lim1* orthologs are known to regulate the migration of differentiating neurons in numerous developmental contexts. This occurs in, for example, motor neurons of the lateral motor columns of vertebrate spinal cords [44,45], horizontal cells of the mouse retina [95], Cajal-Retzius cells of the mammalian cerebral cortex [96], precursor cells of the mouse hippocampus [97], presumptive mitral cells of frog olfactory bulbs [39], and interneurons of nematode head ganglia [90].

## Conclusions

A number of observations of gene expression presented in detail here are consistent with conservation of function of Lhx genes in bilaterian neural development and maintenance. Further work is needed to verify the particular identities of *Narap* and *Narlim1*-expressing neurons. However, based on the strikingly similar activities of *ap* orthologs, and of *lim1* orthologs, in *Drosophila* and vertebrates (for example, [32,36,43,44]), the observed expression of these genes in *Neanthes* nerve cord ganglia likely reflects their control over axonal guidance and bundling (along longitudinal tracts) in separate sets of interneurons, and a portion of the observed *Narlim1* expression in each nerve cord ganglion may well control motor axon targeting to dorsal musculature. Our observations also lead us to infer that *Narap* may promote neuropeptide synthesis in certain sets of interneurons within the nerve cords and posterior brain, and that *Narlim1* may contribute to the initial specification and continued morphogenesis of the anterior brain while controlling the migration of differentiating neurons in the posterior brain.

*Narap* and *Narlim1* appear to be important for parapodial morphogenesis: *Narlim1* expression in parapodial ectoderm suggests a patterning role in the ligules, and the non-overlapping expression of both genes in developing segmental mesoderm correlates with the formation of separate parapodial muscles, suggesting that a complementary LIM code underlies the specification of parapodial myoblasts. Our parapodial expression data, lacking specific similarity to the expression patterns (or absence thereof) of *ap* and *lim1* orthologs in developing arthropod or vertebrate appendages, are consistent with a range of scenarios concerning bilaterian appendage evo-devo. One possibility is independent co-option of Lhx gene function in annelid, arthropod, and vertebrate appendage formation from a shared ancestral neural regulatory network. Alternatively, the dissimilar developmental-genetic patterns may reflect independent divergence of annelid, arthropod, and vertebrate appendages from a common ancestral structure: a presumed sensory outgrowth that possibly arose before the cnidarian-bilaterian split [65,66]. Although the previous scenarios are in conflict over co-option of *Narap* and *Narlim1* in parapodial morphogenesis, our data do seem to indicate less equivocal cases of recruitment of Lhx activity during the evolution of annelid-specific structures; examples include *Narap*’s expression in the jaw sacs and in the presumed Langdon’s organs.

## Methods

### Gene isolation

RNA extraction, cDNA synthesis, and PCR (degenerate PCR for initial gene fragments, RACE PCR for additional 5′ and 3′ fragments) conformed to protocols described in Winchell *et al*. [8]. Initial amplification of a 140 base-pair (bp) fragment of the *ap* homeobox involved the forward primers 5′-acnwsnttyaarcaycaycarct-3′, which target the sequence coding for TSFKHHQL, and the reverse primers 5′-gcrttytgraaccanacytg-3′, which target the sequence coding for QVWFQNA. Initial amplification of a 149 bp fragment of the *lim1* homeobox involved the forward primers 5′-ggnccnmgnacnacnathaargc-3′, which target the sequence coding for GPRTTIKA, and the reverse primers 5′-ckrttytgraaccanacytgdat-3′, which target the sequence coding for IQVWFQNR. Post-PCR purification, cloning, plasmid preparation, sequencing, and assembly of consensus composite sequences followed Winchell *et al*. [8].

### Sequence analysis

We used BLAST [98] to provisionally assign each composite sequence to a LIM subfamily, and to identify homologous sequences for phylogenetic analyses. The amino acid data set for these analyses, aligned according to Winchell *et al*. [8], consisted of the LIM1 (approximately 50 amino acids) and LIM2 (approximately 54 amino acids) domains, the linker between them (approximately 12 amino acids), and the homeodomain (approximately 60 amino acids). We used the alignment of Farfán *et al*. [87] as a guide in delimiting the boundaries of these domains. For optimal tree searches and measurement of nodal support, we used Maximum Likelihood (ML), Bayesian Inference (BI), Maximum Parsimony (MP), and Minimum Evolution (ME) criteria; these were executed according to Winchell *et al*. [8] except for the following modifications. Using PAUP* [99], starting trees for the MP search were obtained via random stepwise addition with 1,000 replicates, and MP non-parametric bootstrapping included 1,000 replicates with 10 random additions per replicate. For the ML search, we implemented the LG + I + G model (determined by ProtTest [100] to best fit the data) in PhyML [101], and used the best PhyML-calculated ML tree as the starting tree for the ML bootstrap analysis, which included 1,000 replicates. For the BI search, we used the JTT + I + G model, as MrBayes [102] does not accommodate the LG model, and the next best model according to ProtTest was JTT. The search included two independent runs, each for two million generations, sampled every 100. Using the sumt command, we discarded the first 2,001 samples as burnin, combined the remaining trees from each run (36,000 total), and calculated a 50% majority-rule consensus tree.

### Whole-mount *in situ* hybridization

We collected, fixed, and stored juvenile *N*. *arenaceodentata* from a laboratory population using the methods of Winchell *et al*. [48]. We synthesized sense and antisense digoxigenin-labeled riboprobes by *in vitro* transcription using SP6 and T7 MEGAscript kits (Life Technologies, Carlsbad, CA, USA) and Dig-11-UTP (Roche Applied Science, Indianapolis, IN, USA). The *ap* riboprobe, 1,241 bases long, contained 141 bases of 5′ UTR and 1,100 bases of open reading frame, including both LIM boxes and the homeobox. The *lim1* riboprobe, 1,002 bases long, contained 204 bases of 5′ UTR and 798 bases of open reading frame, including both LIM boxes. We followed the protocol of Winchell *et al*. [8] for *in situ* hybridization, digoxigenin detection, storage of stained juveniles, and their processing for photomicroscopy. However, in regard to the latter, we added a Hoechst 33342 treatment before the glycerol dehydration: worms were incubated for one hour at room temperature with gentle mixing in a 1 μM solution of this fluorescent DNA label, which was diluted in pure water plus 0.1% Tween 20 (H_2_0Tw). After labeling, we washed the worms three times, each time for five minutes, in H_2_0Tw.

### Immunolabeling

To visualize cilia and neuronal projections, we labeled acetylated α-tubulin in 7- and 8-chaetiger juveniles following the strategy of Winchell *et al*. [48], with a longer Proteinase K digestion (15 minutes total), and a longer first permeablization/blocking wash (extended to 2 hours). The antibody dilutions remained the same, but the secondary antibody was horseradish peroxidase-conjugated goat anti-mouse IgG (Jackson ImmunoResearch, West Grove, PA, USA). To detect this antibody, we incubated the worms in equal parts 1% 3,3′diaminobenzidine (Sigma-Aldrich, St. Louis, MO, USA), 1% nickel chloride, and 0.3% hydrogen peroxide for five minutes at room temperature (these solutions were diluted in pure water). Following the reaction, the worms were washed three times, each time for five minutes in H_2_0Tw, taken through a methanol gradient (five minutes in each of the following methanol solutions in H_2_0Tw: 25%, 50%, 75%, 100%), and stored in 100% methanol at −20°C.

### Histology, photomicroscopy, and image processing

We chose uncontorted, intensely stained worms from the *in situ* hybridization and immunolabeling experiments for transverse sectioning. They were taken from storage, rehydrated into tap water plus 0.1% Tween 20 (by reversing the above methanol gradient steps), counterstained with Nuclear Fast Red (Vector Laboratories, Burlingame, CA, USA) for 10 minutes at room temperature, and then rinsed for 5 minutes in tap water before dehydration and embedding in Poly/Bed 812-BDMA (Polysciences, Warrington, PA, USA) following the manufacturer’s protocol. Sectioning was done on an LKB ultramicrotome at a thickness of 5 μM using a Histo diamond knife (Diatome, Hatfield, PA, USA). Sections were mounted under coverslips in Poly-Mount medium (Polysciences, Warrington, PA, USA) on Tissue Tack slides (Polysciences, Warrington, PA, USA). Whole-mount specimens and sections were analyzed with a Leica DMR microscope using Nomarski, brightfield, or ultraviolet epifluorescence illumination, and photographed with a Nikon Coolpix 4300 digital camera. Image brightness and contrast were adjusted with Adobe Photoshop, and z-projection images were made with ImageJ. We constructed false-colored images of alkaline phosphatase staining by opening a brightfield micrograph in Photoshop, setting the background color to black, using the Select/Color Range command to choose relevant pixels, and then clearing the inverse selection. We applied the red lookup table to this image in ImageJ (after first converting it to 8-bit grayscale), and then optimized its brightness and contrast. Finally, we merged this image and its corresponding Hoechst 33342-fluorescence image into one image using the Screen layer-blending mode in Photoshop.

## Abbreviations

AAT: Acetylated alpha-tubulin; AP: Anteroposterior; Ba: Bayesian posterior probability; bp: Base pair(s); dpf: Days post-fertilization; Lhx: LIM homeobox; LIM-HD: LIM-homeodomain; ME: Minimum Evolution; ML: Maximum Likelihood; MP: Maximum Parsimony; *Nar*: *Neanthes arenaceodentata*; PGZ: Posterior growth zone; RACE: Rapid amplification of cDNA ends; UTR: Untranslated region; VNC: Ventral nerve cord.

## Competing interests

The authors declare that they have no competing interests.

## Authors’ contributions

CJW conceived and designed the study with DKJ, performed all aspects of the research, and wrote the manuscript with help from DKJ. Both authors read and approved the final manuscript.
